# The Pleth Variability Index as a Guide to Fluid Therapy in Dogs Undergoing General Anesthesia: A Preliminary Study

**DOI:** 10.3390/vetsci11090396

**Published:** 2024-08-27

**Authors:** Caterina Vicenti, Noemi Romagnoli, Marzia Stabile, Carlotta Lambertini, Claudia Piemontese, Francesca Spaccini, Armando Foglia, Luca Lacitignola, Antonio Crovace, Francesco Staffieri

**Affiliations:** 1 Section of Veterinary Clinics and Animal Production, Department of Precision and Regenerative Medicine and Ionian Area (DiMePre-J), University of Bari, 70010 Valenzano, Italy; caterina.vicenti@uniba.it (C.V.); marzia.stabile@uniba.it (M.S.); claudia.piemontese@uniba.it (C.P.); luca.lacitignola@uniba.it (L.L.); antonio.crovace@uniba.it (A.C.); 2 Department of Veterinary Medical Sciences, Alma Mater Studiorum-University of Bologna, Via Tolara di Sopra 50, Ozzano Emilia, 40064 Bologna, Italy; noemi.romagnoli@unibo.it (N.R.); carlotta.lambertini2@unibo.it (C.L.); francesca.spaccini@studio.unibo.it (F.S.); armando.foglia2@unibo.it (A.F.)

**Keywords:** fluid therapy, conventional fluid management, pleth variability index, dog, intraoperative management, anesthesia

## Abstract

**Simple Summary:**

The purpose of this study was to evaluate the efficacy of using the pleth variability index (PVi) to guide fluid therapy in dogs undergoing surgery under general anesthesia compared to conventional fixed-fluid-rate administration. Twenty-seven dogs meeting specific criteria were randomly assigned to either a conventional fluid management (CFM) group or a PVi-guided (PVi) group. The CFM group received a fixed rate of fluid, while the PVi group had their fluid rate adjusted based on their PVi values. Results showed that dogs in the PVi group received less total fluid and experienced fewer hypotensive episodes compared to the CFM group. In addition, the mean arterial pressure (MAP) was significantly higher in the PVi group during surgery. These findings suggest that PVi-guided fluid therapy may result in more targeted fluid administration and improve the hemodynamic stability in anesthetized dogs. However, further studies with larger sample sizes are needed to confirm these results and explore the broader applicability of the PVi in veterinary anesthesia.

**Abstract:**

The aim of this prospective, randomized clinical trial was to evaluate the use of the pleth variability index (PVi) to guide the rate of intraoperative fluid therapy compared to a traditional fixed-fluid-rate approach in ASA 1–2 dogs undergoing surgery. Twenty-seven dogs met the inclusion criteria and were randomly assigned to the conventional fluid management group (CFM, *n* = 12) or the PVi-guided group (PVi, *n* = 15). The CFM group received a fixed rate of 5 mL kg^−1^ h^−1^ of crystalloid solution, while in the PVi group the rate was continuously adjusted based on the PVi: PVi < 14% = 3 mL kg^−1^ h^−1^; 14% ≤ PVi ≥ 20% = 10 mL kg^−1^ h^−1^; and PVi > 20% = 15 mL kg^−1^ h^−1^. Hypotension (MAP < 65 mmHg) in the CFM was treated with a maximum of two fluid boluses (5 mL kg^−1^ in 10 min) and in the case of no response, dobutamine (1–3 mcg kg^−1^ min^−1^) was administered. In the PVi group, the treatment of hypotension was similar, except when the PVi > 14%, when dobutamine was started directly. Total fluid volume was significantly lower in the PVI group (0.056 ± 0.027 mL kg^−1^ min^−1^) compared to the CFM group (0.132 ± 0.115 mL kg^−1^ min^−1^), and the incidence of hypotension was lower (*p* = 0.023) in the PVi group (0%) compared to the CFM group (41%). The mean arterial pressure (MAP) was significantly higher in the PVi group during surgery. Dobutamine was never administered in either group. Preliminary data suggest that the PVi may be considered as a potential target to guide fluid therapy in dogs; larger studies are needed, especially in cases of cardiovascular instability.

## 1. Introduction

Intraoperative fluid management is critical to maintaining adequate organ perfusion, with hypovolemia and excessive fluid administration being potential complications [1]. The predominant approach to fluid management involves monitoring parameters such as heart rate (HR), mean arterial pressure (MAP), and central venous pressure (CVP). However, clinical research suggests that changes in MAP are inadequate for assessing stroke volume (SV) and cardiac output (CO), and that CVP measurement alone is insufficient for predicting fluid responsiveness [2,3].

An alternative method is goal-directed fluid management (GDFM), which involves tailored fluid administration using both static parameters (HR, CVP, etc.) and dynamic indicators: stroke volume variation (PPV), arterial pulse pressure variation (PPV) [4], systolic pressure variation (SPV), and plethysmographic waveform variation (PWI). In human medicine, PPV during mechanical ventilation is a robust tool for guiding volume therapy [5,6]. Respiratory variations in the pulse oximeter waveform and pulse reliably predicted fluid responsiveness [7,8]. The pleth variability index (PVi), a dynamic variable that automatically and continuously measures the respiratory variations in the pulse oximeter waveform amplitude, is as effective as stroke volume variation in predicting fluid responsiveness [9]. However, it remains unclear whether optimizing the intraoperative PVi improves fluid management and circulation. Technologies such as Masimo’s Signal Extraction Technology (SET) offer tools such as the PVi to monitor respiratory variations in ventricular preload. The PVi, which is calculated based on the perfusion index (Pi), provides continuous insight into circulatory perfusion and helps clinicians optimize fluid management.

In veterinary medicine, a fixed fluid rate may not be appropriate for all patients because fluid requirements vary based on preoperative conditions, clinical context (dehydration, bleeding, congestive heart failure, and chronic renal failure), and surgical blood loss [10,11]. It is important to investigate whether the PVi can assess fluid status in anesthetized veterinary patients. The PVi demonstrates accuracy in differentiating between fluid responders (patients who respond positively to fluid loading by increasing their CO/SV by 10–15%) [12] and non-responders, aiding in fluid optimization and hypotension prevention. Hypotension in anesthetized patients can be predicted early by the PVi, allowing for preventive measures [13,14]. However, even with normal values of MAP and HR, dogs under general anesthesia may exhibit preload dependence when subjected to a small volume fluid challenge (FC) [15]. This condition can be anticipated by assessing dynamic preload parameters, with PPV and the PVi being more accurate than SPV and SVV. Notably, MAP is a poor predictor of fluid responsiveness (FR), and preload dependence is not necessarily associated with hypotension. A PVi greater than 14% has been shown to be a cut-off value for distinguishing fluid responders from non-responders in dogs [15].

The use of the PVi to guide fluid therapy has not been studied extensively in dogs. The aim of the study was to compare the use of the PVi to guide the rate of intra-operative fluid therapy with conventional fluid management (CFM) in ASA 1–2 dogs undergoing surgery. The cut-off value of the PVi distinguished the dogs requiring increased fluid intake from those in adequately hydrated states conditions. It is hypothesized that the dynamic adjustment of the fluid rate throughout the surgical procedure, guided by the PVi, will prevent or reduce the need for bolus administration and potentially reduce hypotensive events. Endpoints of the study were the total amount of fluid administered, incidence of cardiovascular instability, and use of cardiovascular supportive drugs.

## 2. Materials and Methods

This prospective, randomized, multicenter clinical study was approved by the Ethics Committee for Clinical and Zootechnical Studies on Animals of the Department of Precision and Regenerative Medicine and Jonic Area of the University of Bari, Italy (Prot. n. 1082 III/13), and was conducted in two centers: the University of Bari (Bari, Italy) and University of Bologna (Bologna, Italy). This article is reported in accordance with the Consolidated Standard of Reporting Trials (CONSORT) Statement for the reporting of randomized controlled trials [16].

### 2.1. Animals

After written owner consent was provided, dogs undergoing general anesthesia for surgical procedures were included in the study. Inclusion criteria were as follows: a body weight > 6 kg, an ASA status 1 or 2 (based on history, physical examination, and a complete blood count) [17], and surgical procedures involving the limbs, abdomen, skin, or eyes. The exclusion criteria were as follows: patients with other major diseases, an MAP of lower than 65 mmHg at the beginning of the study, thoracic surgery, major cardiovascular and respiratory diseases, pregnant females, a duration of anesthesia shorter than 30 min, and an impossibility to collect the required data.

Dogs were admitted to the clinic two hours before general anesthesia and housed individually in separate cages. Solid food was withheld for six hours, and water for two hours prior to surgery.

### 2.2. Anesthetic Management

Dogs were premedicated with different protocols (alfa-2 and/or opioids), as chosen by the anesthesiologist prior to the procedure. After aseptic preparation, a catheter was inserted into a cephalic vein and general anesthesia was induced with intravenous (IV) propofol (Proposure 10 mg mL^−1^; Merial), which was administered after at least two minutes of pre-oxygenation via a face mask. The trachea was intubated with an appropriately sized cuffed endotracheal tube and connected to a circular breathing system. Anesthesia was maintained with isoflurane (IsoFlo; Zoetis, Italy) [end-tidal isoflurane percentage (EtISO, Eagle, ID, USA) between 1.1–1.4% [18]] in oxygen (FiO_2_ > 0.8). All animals received lactated Ringer’s solution (B.Braun, Ringer Lattato, B.Braun, Mirandola, Italy) by infusion during the procedure at a rate based on the study protocol. Antibiotic therapy and anti-inflammatory therapy were administered according to the surgical procedure.

During anesthesia, dogs were ventilated in a volume-controlled mode, using a tidal volume (TV) of 12 mL kg^−1^ with an inspiratory to expiratory ratio of 1:2, with a 25% end-inspiratory pause. Plateau airway pressure was maintained between 8 and 10 cm, with H_2_O adjusting the TV. Respiratory frequency was titrated based on the end-tidal CO_2_ (EtCO_2_), targeting a value between 35 and 45 mmHg, obtaining an adequate level of anesthesia (absence of palpebral reflex, ventral–medial rotation of the eye, and loss of the jaw tone), and confirming the absence of patient–ventilator asynchrony by a pressure/volume loop and capnography observation.

Monitoring during general anesthesia included peripheral hemoglobin oxygen saturation (SpO_2_; %), assessed with a pulse oximetry probe on the tongue, heart rate (HR; beats/min) obtained from lead II electrocardiography, and noninvasive arterial blood pressure (systolic, diastolic, and mean arterial blood pressure: SAP, DAP, and MAP, respectively, mmHg) using an oscillometric method (Suntech^®^ Vet20, Morrisville, NC, USA). Good agreement between IBP and NIBP has been demonstrated for MAP measurements [19]. The metacarpal arteries were occluded by placing a cuff on the distal part of the thoracic limb, situated between the carpal and metacarpal pads [20] at intervals of five minutes. The width of the cuff was 30–40% of the circumference of the limb at the site of cuff placement, according to the ACVIM consensus statement [21]. In addition, the following were assessed: core body temperature (T; °C) was monitored with an esophageal probe, respiratory rate (RR, breaths/min), EtCO_2_, and FiO_2_ were assessed with a multiparametric monitor, and the PVi and PI were assessed with a Massimo rainbow SET^®^ pulse oximeter. At the end of the procedure, the administration of isoflurane was stopped, and the dogs were disconnected from the circuit when chest expansion and oxygen saturation were satisfactory. The orotracheal tube was removed when the swallowing reflex returned.

### 2.3. Study Protocol

Dogs were randomized into two groups, conventional fluid management (CFM) and PVi-guided fluid therapy (PVi), using a sequence generator available at http://www.random.org/.

For the study, hypotension was defined as a condition in which the MAP was less than 65 mmHg.

The fluid rate was started at 5 mL kg^−1^ h^−1^ from the induction of anesthesia and then adjusted at the start of the study for each dog, based on the study group.

In the CFM group, the fluid rate was maintained at 5 mL kg^−1^ h^−1^ throughout the procedure. In the event of hypotension, a crystalloid bolus of 5 mL kg^−1^ was administered intravenously over 10 min; if hypotension persisted, a second bolus of fluid was administered over 5 min. If hypotension persisted, a constant-rate infusion of dobutamine (2–5 mcg kg^−1^ min^−1^) was administered until the MAP exceeded 65 mmHg. If bradycardia occurred, atropine (40 mcg kg^−1^ IV) was administered.

In the PVi group, a pulse oximetry probe (LNCS TC-I^®^; Masimo Corp., Irvine, CA, USA) was placed on the tongue for all patients. It was securely wrapped to prevent interference from external light sources. The pulse oximeter was connected to the Masimo Radical 7 monitor (Masimo SET; Masimo Corp., USA), which included the PVi software (version 7.0.3.3). The PVi automatically and continuously calculates respiratory variations in the photoplethysmogram using data obtained noninvasively from a pulse oximetry sensor. The PVi reflects the amplitude of the pulse oximeter waveform and is computed by indexing the pulsatile infrared signal (AC or variable component) to the non-pulsatile infrared signal (DC or constant component).

The calculation of the PVi involves the perfusion index (PI)

PI (%) = (AC∕DC) × 100

PVi = [(PImax − PImin)/Pimax] × 100

The perfusion index represents the status of peripheral perfusion and gives information about the strength of the pulse signal at the site of measurement; it has a wide range (0.02% to 20%).

**Figure 1:**
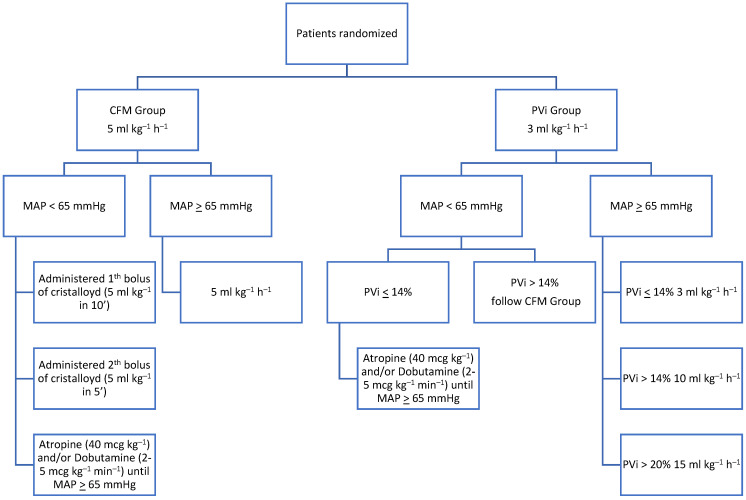
Study flow chart. CFM (conventional fluid management), PVi (pleth variability index), and MAP (mean artery pressure).

After the stabilization of mechanical ventilation, for patients of this group, the infusion of lactated Ringer’s solution was adjusted to 3 mL kg^−1^ h^−1^. If the patient was not hypotensive and the PVi was higher than 14% for more than five minutes, the fluid rate was increased to 10 mL kg h. If the PVi was greater than 20%, the fluid rate was further increased to 15 mL kg^−1^ h^−1^. If the PVi was less than or equal to 14%, the fluid rate was restored at 3 mL kg h. In the case of hypotension, if the PVi was less than or equal to 14%, a constant-rate infusion of dobutamine (2–5 mcg kg^−1^ min^−1^) was administered until the MAP became higher than 65 mmHg. In the case of concurrent bradycardia, an intravenous bolus of atropine (40 mcg kg^−1^) was administered directly; if the PVi was above 14%, the same protocol was followed as in the CFM-group (Figure 1).

In each group, dobutamine infusion was stopped when the MAP was higher than 80 mmHg. In case of the MAP > 100 mmHg, the study was stopped, and cases were managed according to clinical judgment.

The time of the end of the study was recorded and identified after the end of surgery and before the dog was weaned from the MV.

Physiological parameters were collected every five minutes for the entire duration of the study. Only the parameters recorded at the beginning (T0), middle (T1/2), and end (Tend) of the study were considered for data analysis. The number of hypotensive episodes and the total amount of christalloids, dobutamine, and atropine administered during the study time (T0–Tend) were recorded in each case. The total amount of fluid was normalized for BW and infusion time to determine the average fluid rate (mL kg^−1^ min^−1^) for each group. The same was calculated for dobutamine and atropine. The duration of surgery and anesthesia was also recorded.

### 2.4. Statistical Analysis

A statistical analysis was performed using MedCalc 12.7.0.0 software. The normal distribution of the data was confirmed by the D’Agostino test. Mean/median, standard deviation (SD), and 95% confidence intervals (CI) were calculated for all data. Data on age, body weight, total fluid amount, average fluid rate, and the duration of anesthesia were compared between groups using the one-way ANOVA test. The values of the HR, SAP, MAP, DAP, RR, T, PVi, PI, and SpO_2_ were analyzed by a two-way ANOVA test (time and treatment), using the values obtained at T0, T1/2, and Tend. The Tukay test was used for the post hoc analysis. The incidence of hypotension was compared between the groups by a chi-squared test. A *p* value < 0.05 was considered statistically significant.

## 3. Results

**Figure 2:**
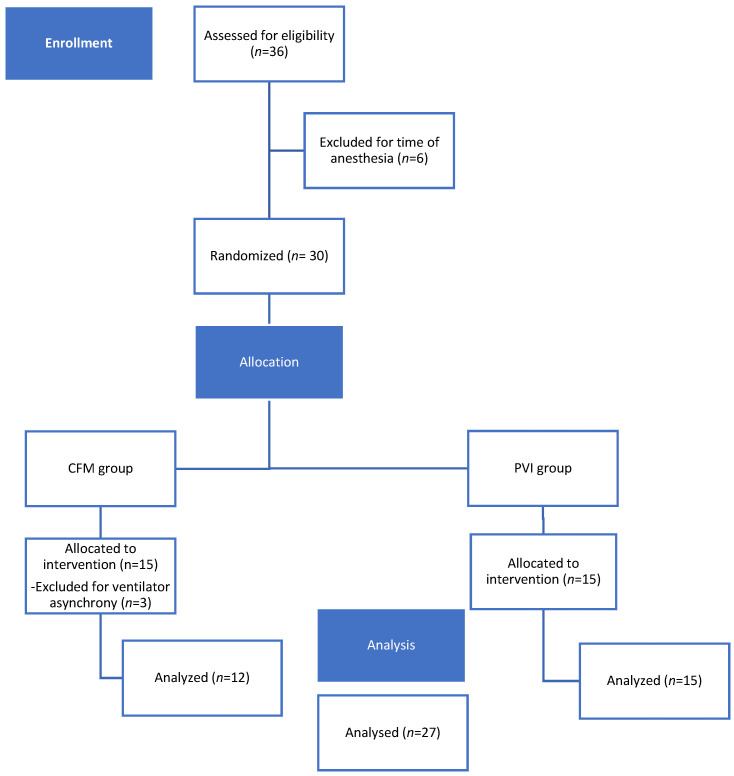
CONSORT flow diagram of the patient enrolment and group allocation.

Thirty-six dogs were evaluated for their eligibility at Center 1 and Center 2. Six cases were excluded because they did not meet the inclusion criteria (duration of anesthesia). Of the 30 dogs enrolled in the study, three were excluded because of asynchrony with the ventilator. Twenty-seven dogs completed the study without any complications. Details regarding the allocation of dogs to the two groups are shown in Figure 2.

**Figure 3:**
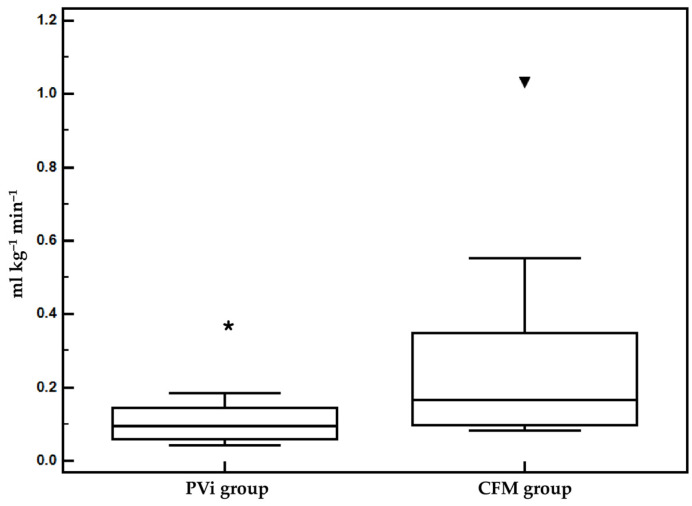
Mean and standard deviation values of the average fluid rate administered in the CFM and PVi groups from T0 to Tend. * *p* < 0.05 versus CFM group.

**Figure 4:**
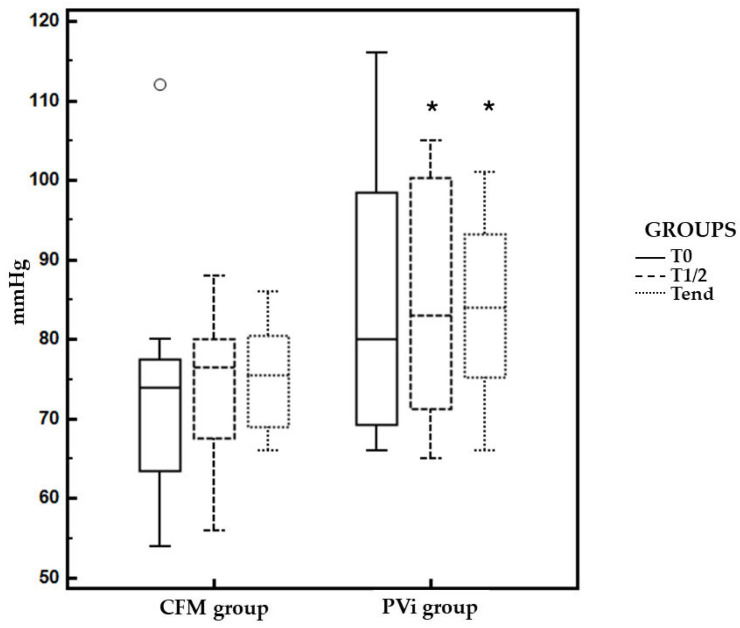
Mean and standard deviation values of mean arterial pressure (MAP) in the CFM group and PVi group from at T0, T1/2 and Tend. * *p* < 0.05 versus CFM group.

The demographic and preoperative characteristics were similar in both groups (Table 1). The duration of anesthesia and surgery were also similar. The average fluid rate was statistically significantly higher in the CFM group (0.132 ± 0.115 mL kg^−1^ min^−1^) compared to the PVi group (0.056 ± 0.27 mL kg^−1^ min^−1^) during the study time (T0–Tend) (Figure 3). The duration of the anesthesia and surgery, the PVi study, and the value of MAP at T0 were similar in both groups (Table 2). The incidence of hypotension was 41.6% in the CFM group and 0% in the PVi group (*p* = 0.023). The MAP was significantly higher at T1/2 and Tend in the PVi group compared to the CFM group (*p* < 0.05) (Table 3 and Figure 4). Dobutamine and atropine were not used in either group.

## 4. Discussion

The results of the present study showed that PVI-based intraoperative fluid therapy can provide better hemodynamic conditions (a lower incidence of hypotension and higher MAP values) with lower average total fluid administration compared to traditional fluid management.

Fluid management during anesthesia and surgery remains a highly controversial topic. Perioperative morbidity has been associated with the amount of intravenous fluid administered, with both inadequate and, more commonly, excessive fluid administration leading to an increase in postoperative complications [22]. Intraoperative fluid administration based on a generalized equation based on body weight per unit time, modified according to the specific needs of the animal and the surgical procedure [23], is not supported by known physiological principles.

Insufficient fluid therapy can lead to various perioperative complications, including low blood pressure, acute kidney injury, irregular heartbeats, and tissue hypoperfusion/ischemia. Conversely, excessive fluid intake may result in a prolonged need for mechanical ventilation, delayed wound healing, an increased risk of infection, and a prolonged hospital stay due to fluid overload [24,25].

Several variables could impact the cardiovascular status of a dog during general anesthesia, such as the types of anesthetic agents administered, body position, and the specific surgical and anesthetic techniques used [26,27]. In this study, the administration of propofol and isoflurane may have caused vasodilation, which may have resulted in a slight reduction in stressed venous blood volume. Consequently, this could lead to a decrease in SV and cardiac index (CI), shifting the heart toward the steeper segment of the Frank–Starling curve. In addition, the use of intermittent positive pressure ventilation (IPPV) may exacerbate this condition [28,29]. Suboptimal SV and CI values are likely responsible for inadequate oxygen delivery (DO_2_), as suggested by previous human studies. Optimizing DO_2_ during surgery has been proposed to improve patient outcomes [30].

Therefore, it could be hypothesized that monitoring dynamic preload indices during anesthesia could provide the prevention of hemodynamic instability, even in cases not associated with alterations in classical parameters (e.g., MAP and HR), and could be a valid tool to guide fluid therapy [26,28].

MAP is usually considered the reference hemodynamic parameter during anesthesia, with the goal of maintaining an adequate hemodynamic state. It is the main cardiovascular parameter that guides fluid therapy and the use of sympathomimetic drugs. However, it is well known that this parameter has several limitations in terms of providing an effective hemodynamic picture of the patient [31]. A recent study in dogs proved that MAP has the lowest accuracy in predicting fluid responsiveness in dogs as compared to other dynamic hemodynamic parameters (SVV, PPV, and the PVi) in dogs [15]. The compensatory mechanisms related to myocardial contractility and peripheral resistance may compensate for the MAP even in patients with an absolute or relative fluid deficit [32].

The preliminary results of this study demonstrate that PVI monitoring may be helpful in identifying patients in need of fluid to prevent the occurrence of hemodynamic instability. In addition, its dynamic nature makes it an ideal tool for adjusting the fluid rate based on patient and/or surgical needs. Using a multimodal approach to hemodynamic monitoring may help to prevent complications related to both hypotension and inappropriate fluid management. In our study the PVi approach resulted in no hypotensive events; in contrast, in the CFM group, hypotension occurred in 41% of cases, requiring additional fluid administration and resulting in a higher average fluid rate. This result may have a very important impact on the clinical management of fluids in the perioperative period. The reference values of the PVi used in this study were taken from Skouropoulou et al., in which 14% was identified as the cutoff value for fluid responsiveness [15]. In addition, the limit of 20% of the PVi to increase the fluid rate was arbitrary chosen considering the direct correlation observed between the PVi and the entity of responsiveness (variation of SV) in dogs without any major systemic disease. Indeed, we may suppose different thresholds in dogs with cardiovascular or renal pathologies. The MAP was significantly higher during surgery in the PVi group, further supporting the effectiveness of PVi-guided fluid therapy in maintaining adequate blood pressure. None of the cases presented significative morbidity during the procedures.

Among all other dynamic hemodynamic parameters, the PVi is by far the less invasive and is more convenient in terms of cost [33]. Several veterinary and human studies have demonstrated its accuracy compared to invasive methods [15]. The PVi is only valid during mechanical ventilation and therefore cannot be used in spontaneously breathing cases. The PVi requires a minimum of 1% of the PI to produce a reliable value, which may be the main limitation of this technology [34], so in dogs with low peripheral perfusion or minimal changes in pulse volume might be less accurate than other hemodynamic indices such as SVV and PPV.

In humane medicine, the use of the PVi for goal-directed fluid therapy (GDFT) management has been investigated in several studies. The PVi seems to guide GDFT similarly to PPV regarding the hospital length of stay, amount of fluid, and incidence of postoperative complications [35,36,37]. Our results are consistent with a recent study in pediatric patients with a protocol very similar to this study [38].

This study has some limitations. Firstly, is the preliminary nature and the simple size, necessitating larger-scale studies to confirm the results. The study focused on ASA 1–2 dogs undergoing surgery, limiting the applicability to more diverse patient populations. One significant limitation is the use of NIBP monitoring rather than invasive blood pressure (IBP) monitoring. NIBP is generally less accurate and less sensitive to rapid changes in blood pressure compared to IBP. As a result, the study’s ability to describe hemodynamic instability is somewhat constrained, potentially leading to an underestimation of the critical fluctuations in blood pressure during the perioperative period. Moreover, it was observed from the collected data that the baseline blood pressure in the PVi group was higher than in the CFM group. Although this difference was not statistically significant, it could have potentially impacted the study’s sensitivity when comparing the two groups. This discrepancy might have introduced some bias, which should be considered when interpreting the results. Additionally, the duration of anesthesia and surgery varied among patients, which could affect fluid requirements and hemodynamic stability. Furthermore, the premedication protocol was not standardized, which could have resulted in different hemodynamic effects depending on the molecules used.

## 5. Conclusions

The preliminary results of this study support the potential utility of PVi-guided fluid therapy in dogs undergoing general anesthesia. Larger, multicenter studies are warranted to validate these findings and explore the broader applicability of the PVi in clinical practice. Further research should also investigate the optimal PVi thresholds and fluid management protocols to maximize patient outcomes while minimizing complications.

In conclusion, this study highlights the potential benefits of incorporating dynamic indicators such as the PVi into intraoperative fluid management strategies, paving the way for more individualized and effective patient care in veterinary anesthesia.

## Figures and Tables

**Table 1:** The demographic and clinical characteristics of dogs enrolled in the study and randomized to the PVi and CFM groups. Data are presented as mean, standard deviation, and 95% confidence intervals (CI).

	CFM-Group (*n* = 12) (95% CI)	PVI-Group (*n* = 15) (95% CI)	*p*
Sex (female/male; *n*)	7/5	5/10	0.212
Age (months)	42.5 ± 34.25 (20.73–64.26)	85.66 ± 37.71 (64.78–106.55)	0.005 *
Body weight (kg)	35.22 ± 13.77 (26.47–43.97)	29.13 ± 13.13 (21.86–36.40)	0.252

* *p* < 0.05.

**Table 2:** Mean, standard deviation, and 95% confidence intervals (CI) of mean arterial pressure (MAP), duration of anesthesia, duration of surgery, duration of the PVI study, and amount of fluid administered in the dogs enrolled in the study and randomized to the PVi or CFM groups.

	CFM-Group (95% CI)	PVI-Group (95% CI)	*p*
MAP at the beginning (mmHg)	85.16 ± 19.74	94.2 ± 23.92	0.303
Duration of anesthesia (min)	151.66 ± 86.21 (96.88–206.44)	134.66 ± 66.24 (97.98–171.35)	0.562
Duration of surgery (min)	76.25 ± 82.11 (24.07–128.42)	53.66 ± 38.27 (32.47–74.86)	0.351
Duration of PVi study (min)	82.08 ± 62.33 (42.48–121.68)	73 ± 41.78 (49.86–96.13)	0.654
Amount of fluids (mL kg^−1^ min^−1^)	0.132 ± 0.115 (0.058–0.20)	0.056 ± 0.27 (0.04–0.07)	0.022 *

* *p* < 0.05.

**Table 3:** Mean, standard deviation, and 95% confidence intervals (CI) of heart rate (HR), systolic, mean, and diastolic (SAP, MAP, DAP) arterial pressure, respiratory rate (RR), the pleth variability index (PVI), and perfusion index (PI) in the dogs enrolled in the study and randomized into PVi or CFM groups. Data were collected at the beginning (T0), in the middle (T1/2), and at the end (Tend) of the study; these time points are not representative of the specific variance of the parameters throughout the procedure. The cardiovascular parameters shown are indicative of the overall performance of the case regardless of the specific variation in blood pressure. Hypotension was treated immediately during anesthesia, as suggested by the protocol.

	CFM-Group (95% CI)	PVI-Group (95% CI)	*p*
HR (beats/min)			
T0	76.41 ± 26.56 (59.53–93.29)	83.26 ± 19.62 (72.40–94.13)	0.443
T1/2	78.25 ± 25.88 (61.80–94.69)	70.13 ± 13.17 (72.40–94.13)	0.296
Tend	85.08 ± 25.51 (68.87–101.29)	74.86 ± 13.22 (67.54–82.18)	0.197
SAP (mmHg)			
T0	109.91 ± 17.54 (98.76–121.06)	119.71 ± 17.95 (109.78–129.67)	0.164
T1/2	108.41 ± 17.40 (97.36–119.47)	115.86 ± 16.15 (106.91–124.81)	0.262
Tend	115.83 ± 16.40 (105.41–126.25)	115.6 ± 10.73 (109.65–121.54)	0.963
MAP (mmHg)			
T0	73.25 ± 15.11 (63.64–82.85)	85.13 ± 16.67 (75.90–94.36)	0.067
T1/2	74.083 ± 9.2 (68.22–79.94)	84.66 ± 14.70 (76.52–92.81)	0.039 *
Tend	75.167 ± 6.68 (70.91–79.41)	84 ± 10.13 (78.38–89.61)	0.015 *
DAP (mmHg)			
T0	61.08 ± 12.24 (53.30–68.86)	70.93 ± 14.94 (62.65–79.21)	0.078
T1/2	66.08 ± 15.79 (56.04–76.12)	71.6 ± 15.58 (62.96–80.23)	0.372
Tend	66.58 ± 14.89 (57.11–76.04)	69.8 ± 12.13 (63.08–76.51)	0.542
RR (breaths/min)			
T0	14.5 ± 2.1106 (13.159–15.841)	13.333 ± 3.2878 (11.513–15.154)	0.297
T1/2	15.333 ± 3.6013 (13.045–17.622)	13 ± 2.9277 (11.379–14.621)	0.074
Tend	15.583 ± 4.358 (12.814–18.352)	13.333 ± 2.9921 (11.676–14.990)	0.124
PVi (%)			
T0	15.33 ± 5.80 (11.64–19.02)	17.53 ± 7.94 (13.12–21.93)	0.431
T1/2	12.75 ± 4.57 (9.84–15.65)	15.2 ± 7.32 (11.14–19.25)	0.322
Tend	12 ± 6.09 (8.13–15.87)	11.8 ± 4.24 (9.44–14.15)	0.921
PI			
T0	1.8 ± 1.6 (0.74–2.85)	1.65 ± 1.50 (0.78–2.52)	0.817
T1/2	1.87 ± 1.19 (1.11–2.63)	1.63 ± 0.94 (1.08–2.18)	0.573
Tend	1.52 ± 0.97 (0.83–2.06)	1.31 ± 0.91 (0.78–1.84)	0.584

* *p* < 0.05.

## Data Availability

The data are available by contacting the authors.
